# Fetal MRI as Complementary Study of Congenital Cystic Adenomatoid Malformation During Pregnancy: A Single Case Report

**DOI:** 10.7759/cureus.570

**Published:** 2016-04-15

**Authors:** Jose Martinez-Mas, Alberto Miranda-Paanakker, Paloma Gomez-Leal, Patricia Navarro-Sanchez, Andres Bueno-Crespo, Juan Pedro Martinez-Cendan, Manuel Remezal-Solano

**Affiliations:** 1 Obstetrics and Gynecology Department, Santa Lucía University Hospital, Cartagena, Murcia, Spain; 2 Radio-Diagnostic Service, Santa Lucía University Hospital, Cartagena, Murcia, Spain; 3 Department of Computer Science, Catholic University of Murcia (UCAM), Murcia, Spain; 4 Obstetrics and Gynecology Department, Catholic University of Murcia (UCAM), Murcia, Spain

**Keywords:** fetal magnetic resonance imaging, fetal mri, congenital cystic adenomatoid malformation, ccam, fetal lung mass, differential diagnosis

## Abstract

Fetal lung masses are rare findings in prenatal ultrasound scanning in general population, of which congenital cystic adenomatoid malformation is the most commonly diagnosed type. This paper reports a single case of congenital cystic adenomatoid malformation detected at our hospital and the subsequent clinical follow-up using ultrasound scanning and fetal magnetic resonance imaging.

## Introduction

Fetal lung masses are thought to occur in one out of every 15,000 live births, but the real incidence is likely higher because of the development of higher accuracy prenatal ultrasound scanning and its widespread application [1]. Even with an experienced ultrasound specialist, there are some limitations to ultrasound scanning and this could require the use of other complementary techniques such as fetal magnetic resonance imaging (MRI). Lung masses can be cystic or solid, representing a wide array of developmental malformations including congenital cystic adenomatoid malformation, bronchopulmonary sequestration, bronchogenic cyst, congenital lobar emphysema and segmental bronchial atresia [2-3].

Congenital cystic adenomatoid malformation is the most commonly diagnosed lung mass in newborn children. It used to be classified as a lung hamartoma characterized by terminal bronchial proliferation and the absence of normal alveoli. Its blood supply comes through pulmonary arteries and drains towards pulmonary veins. This lesion is usually diagnosed after the 20th week of pregnancy, by ultrasound scanning, where a pulmonary echogenic cystic or solid mass can be observed. Sometimes they can be misdiagnosed as diaphragmatic hernia or vice versa. The natural course of these lesions depends on the size of the mass and the physiologic developments secondary to compression of nearby structures. The growth can occur from 18 to 26 weeks of gestation reaching a stable or sometimes decreased size at term, when compared to the overall size of the fetus [1].

Most cases will be asymptomatic in fetal and postnatal periods, except for those that compress the mediastinum, the vena cava or the heart, impairing proper right atrial blood return. In these cases the fetus will develop heart failure, hydrops and, unless treated, will end in death. Hydrops fetalis presence is the main prognostic sign [4]. Sometimes smaller-sized lesions can grow later than expected, therefore it is essential to perform a close follow-up. Typically, lung masses smaller than 57% volume of the total lung volume tend to resolve completely. Those with a volume bigger than 84% usually have an incomplete resolution. Treatment options of these large masses include maternal administration of steroids, thoracocentesis or thoracoamniotic shunt and open fetal surgical resection. This last procedure has been successfully performed only in two centers. Percutaneous intrauterine laser therapy has been published as another option too [4-6].

These lesions can be of multiple histologic varieties, but most of them will be benign. However, differential diagnosis with pulmonary blastoma, a rare kind of pediatric lung cancer [7], has to be done. If the lesions are large enough, newborns can develop respiratory distress as the lesion gets filled with air and expands, shifting the mediastinum and compressing the surrounding lung tissue. The majority of patients, especially those with segmental lesions, are asymptomatic, but the mass should be resected during neonatal period due to its higher risk of developing recurrent infections.

There isn't much objective data that allows to predict the outcome during prenatal follow-up. Crombleholme et al. [8] were the first group to try to risk-stratify fetuses depending on the size of their lung masses. This group developed the congenital cystic adenomatoid malformation volume ratio (CVR), in which the volume of the lung malformation is estimated by the formula for a prolate ellipse and then divided by head circumference to correct for overall fetal size and gestational age. In their series, they found a cut-off of 1.6 where those fetuses with initial CVR over 1.6 had a higher rate of hydrops, need for fetal intervention, and fetal or postnatal death. In this manner, Cass et al. [1] performed a review of a bigger series of fetuses with lung masses to further correlate prenatal findings with fetal and postnatal outcomes, finding 2.0 as the single most useful threshold regarding hydrops, heart failure, fetal intervention, mortality and fetal intervention.

In this paper we report a single case detected and followed-up at our hospital, located in Cartagena, a coastal city in southeastern Spain with 216,000 inhabitants, where we assist around 2,900 deliveries per year. The patient was referred in 2015 from her private gynecologist to our center in her 28th week of gestation suspecting a congenital cystic adenomatoid malformation.

## Case presentation

A 41-year-old woman, in her second pregnancy after a cesarean section in 2009 (due to a cervical myoma that impeded a vaginal delivery) presented to our hospital. We obtained verbal consent from her to use the information from her case for teaching and research purposes.

**Figure 1:**
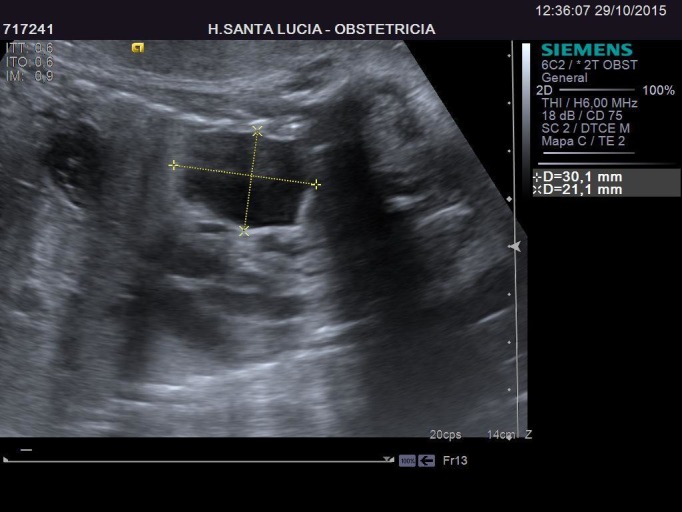
Ultrasound - Sagittal View This figure shows the size of the congenital adenomatoid malformation. Notice that the stomach is in the abdomen, below the thorax.

**Figure 2:**
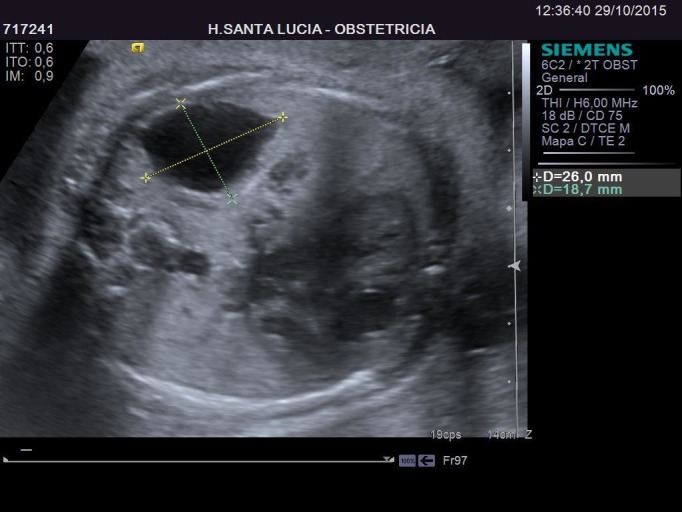
Ultrasound - Transversal View This figure shows the size of the congenital adenomatoid malformation.

**Figure 3:**
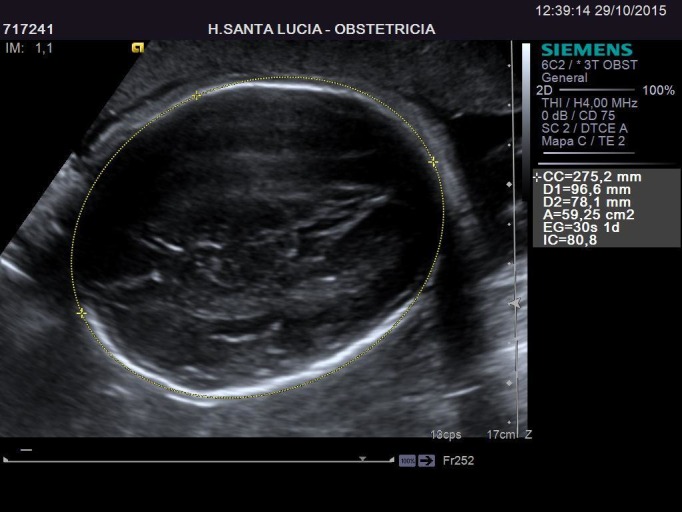
Cephalic Circumference

In her first trimester screening, an intermediate risk (1/565) for Down Syndrome was obtained. A Harmony test was performed with a normal result. In the 20th week of pregnancy her morphological scan was normal. In the 27th week, during a routine scan, a large cystic image appeared, occupying about half the size of the left lung of the fetus. This image was not visible four weeks earlier. The first impression suggested a congenital cystic adenomatoid malformation, but it also resembled a left diaphragmatic hernia, with the stomach occupying part of the left lung field just behind the heart, and slightly misplacing it to the right. The heart structure and anatomy was strictly normal. After calculating CVR (0.37), using the diameters (Figures 1-2) and cephalic circumference (Figure 3), it was determined that the risk to develop hydrops fetalis was 5%.

**Figure 4:**
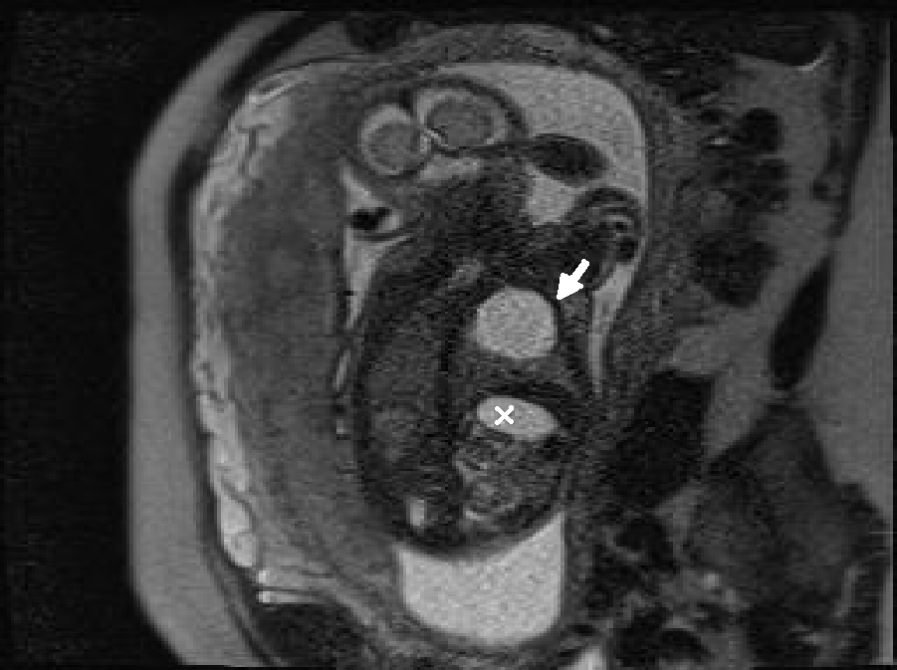
HASTE T2 Coronal Orientation This figure differentiates gastric (x) and cystic (white arrow) chambers showing liquid with hyperintense signal.

**Figure 5:**
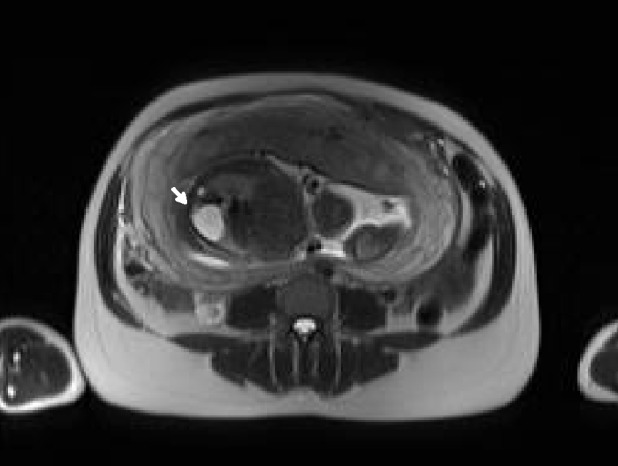
HASTE T2 Axial Orientation This figure shows CCAM (white arrow) located inside left lung structure. CCAM - congenital cystic adenomatoid malformation.

**Figure 6:**
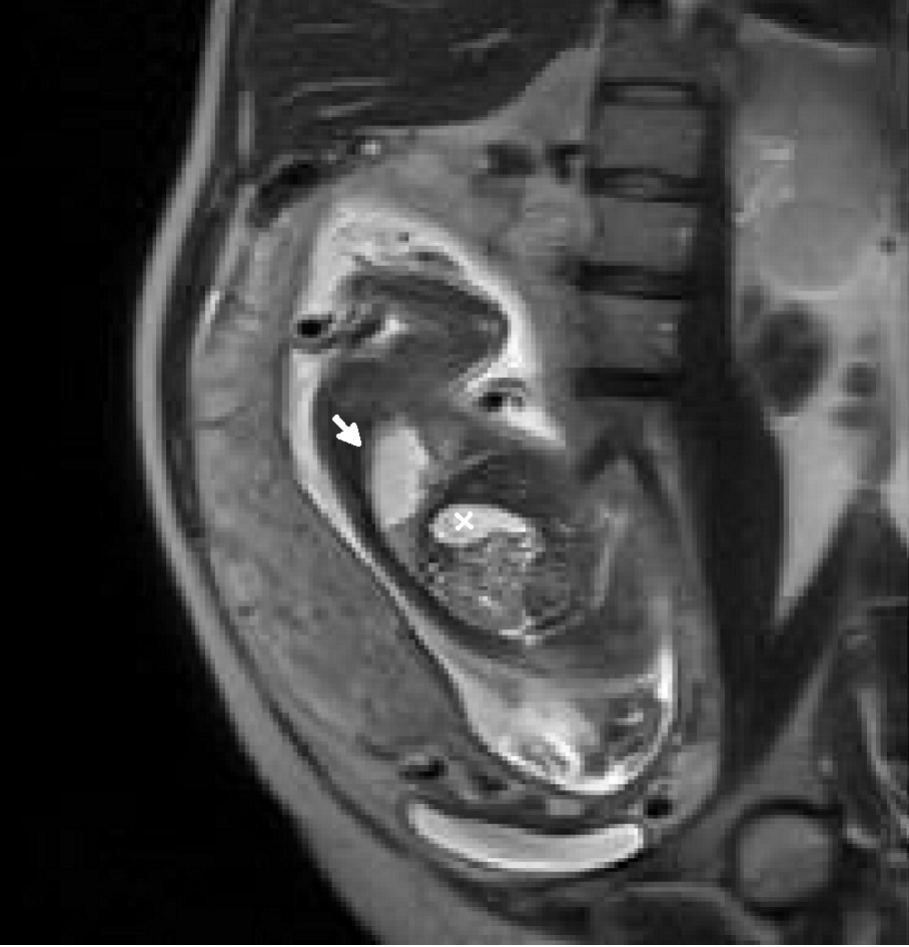
HASTE T2 Sagittal Orientation In this figure, the diaphragm is shown clearly between the CCAM (white arrow) and the stomach chamber (x).

**Figure 7:**
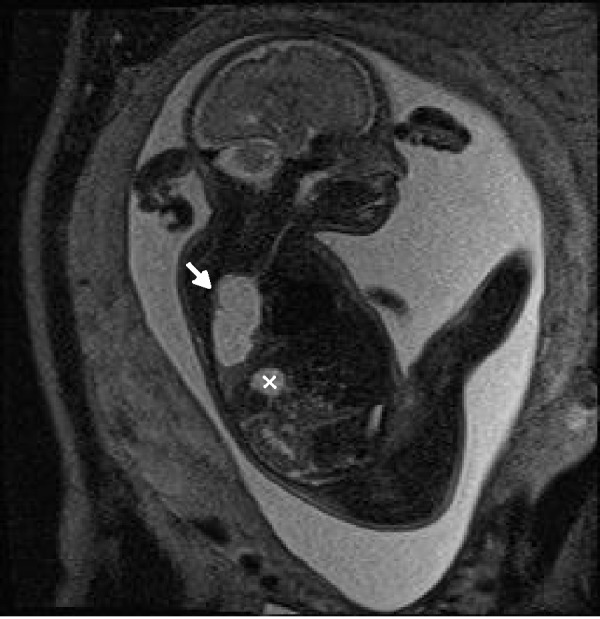
HASTE T2 Fat Suppressed This feature shows CCAM (white arrow) separated from the gastric chamber (x) by the diaphragm.

**Figure 8:**
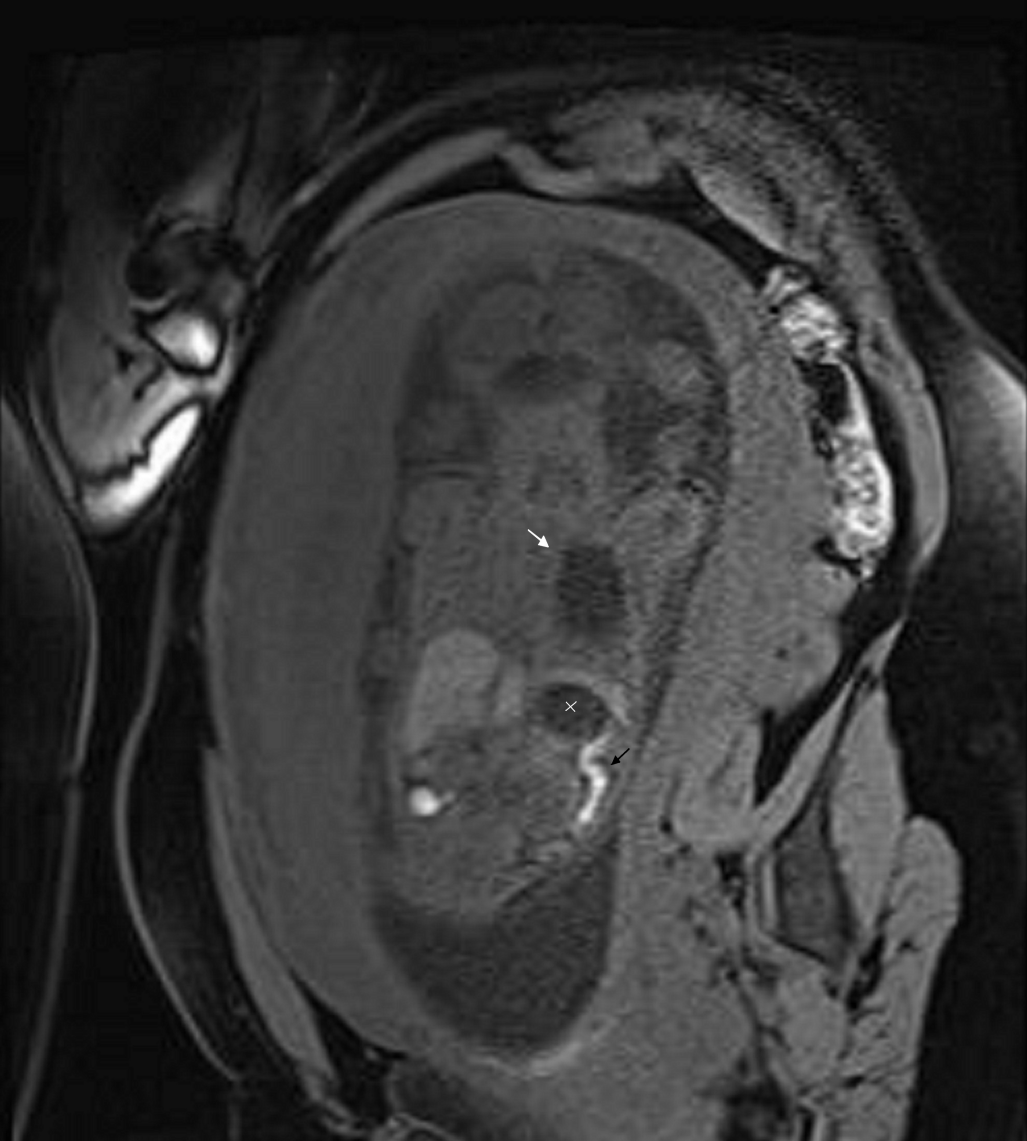
VIBE FS Coronal Orientation This figure helps to differentiate CCAM from diaphragmatic hernia, showing gastric (x) and cystic (white arrow) chambers with low-intensity signal and meconium (black arrow) with hyperintense signal.

An amniocentesis was performed, and two days later an MRI was done in order to confirm the diagnosis. Fetal MRI was performed using high field resonances (Siemens 1.5T, Erlangen, Germany) with high spatial resolution multichannel coils. The main features used are breath-holding by the mother, half-Fourier acquisition single-shot turbo spin-echo (HASTE) weighted on T2, fetal coronal, axial and sagital T2 HASTE TE 168 ms orientations, fetal transversal T2 HASTE fat-suppressed (FS), and fat-suppressed 3D gradient echo volume interpolated breath-hold examination (VIBE FS) with coronal and transversal orientation [9]. Figures 4-8 show representative images from this patient in different features studied.

The fetal MRI was reported as type I congenital cystic adenomatoid malformation (Stocker classification) vs bronchogenic cyst. It had a 27 x 26 x 19 mm overall size, and was slightly pushing the mediastinum and the heart to the right. The total lung volume calculated was 32 cc, and lung mass volume was 16 cc. As it was less than 57% of total lung volume, this lesion could be fit for a complete resolution after birth [9].

The patient underwent a scan every two weeks in our Fetal Medicine Unit. It was found in week 31 that the lung mass was 43 x 28 x 22 mm, without cardiac displacement, and that the fetal estimated weight was in the 97th percentile. The heart had a normal structure, but a small pericardiac leak was found under the AV valves (3.4 mm). Heart function was preserved during follow-up. The rest of the study was normal. The placenta had a previous-occlusive insertion.

**Figure 9:**
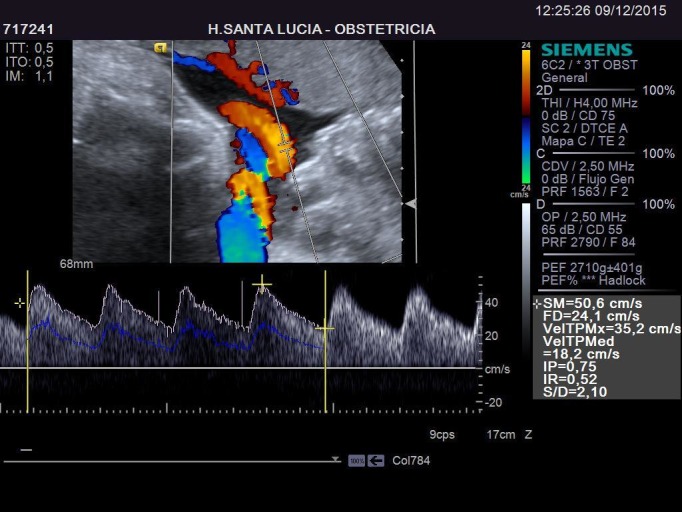
Umbilical Artery Doppler in Week 35

**Figure 10:**
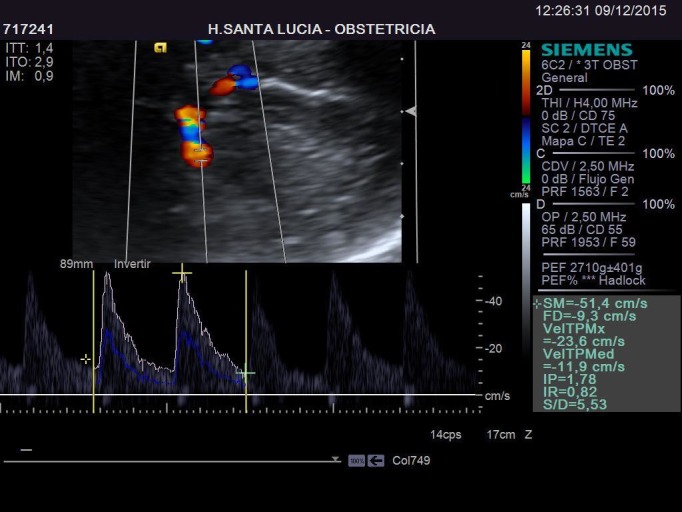
Middle Cerebral Artery Doppler in Week 35

The patient had a glucose intolerance (positive O'Sullivan test), therefore she had a glucose tolerance test with 100 g glucose, which resulted negative for gestational diabetes. In the 33rd week scanning, the lung mass size was 39 x 25 x 20 mm and fetal estimated weight was in the 83rd percentile. At this point, the patient decided she would give birth at another hospital, closer to her home. The last visit to our hospital was in week 35. The size of the cyst was 42 x 29 x 24 mm. It had about the same size compared to previous scans correlating its size to the fetus head circumference. There wasn't any sonographic sign that suggested heart disfunction or failure and it is evident in the Doppler fetal study in Figure 9 and Figure 10.

## Discussion

This is not a frequent case in our area and an interdisciplinary approach can offer different points of view during the study. In our experience, MRI has helped us to distinguish between a suspicious diaphragmatic hernia and a demonstrated lung malformation, and to plan the follow-up process.

In our hospital, the experienced radiologists in fetal MRI were the key professionals who treated these type of cases. On the other hand, we found that it is essential to have an obstetrical team specialized in fetal medicine that would allow us to perform a close follow-up of the patient and the malformation. Ultrasound scanning still remains the basic tool to follow these patients, and in these cases, enables the calculation of an accurate CVR as a starting point to risk-stratify the patient.

As a multidisciplinary group of medical specialists involving obstetricians, perinatologists, radiologists, midwives, nurses, etc, we have a weekly meeting where all patients seen at our unit are discussed and decisions concerning their management are made.

We agree with other authors [7, 9] on the key role of imaging techniques in prenatal detection of these malformations, and the need for a close follow-up of the patient during pregnancy and after birth, in order to make the right diagnosis and to provide proper treatment [10].

We won't be able to follow the final stage of pregnancy of our patient due to her personal interests; however, we would like to share our clinical findings thus far. We will also keep in touch with our colleagues and other specialists involved in the future management of our patients, concerning both mother and newborn.

## Conclusions

Prenatal detection of lung masses is increasing due to the great development in imaging technology and its widespread use. Most of prenatally diagnosed pulmonary abnormalities will not require immediate intervention. However, imaging evaluation and close clinical follow-up during pregnancy and after birth are of critical importance to confirm the diagnosis and decide the adequate treatment.

The baby was born during the development of this paper. The patient checked in due to amniorrhea, and a cesarean section was performed, because of the risk of fetal hypoxia. The newborn had an APGAR score 9/10/10, and the umbilical cord blood pH was 7.31 and 7.27. Two months after the delivery, the newborn is still asymptomatic, and a high resolution computerized axial tomography is pending.

We are interested in the newborn’s development and especially in the possibility of complete resolution after birth of the congenital cystic adenomatoid malformation, as suggested by the volume of the mass during the follow-up. It would be interesting to have the final after-birth diagnostic; but this process will take a few months to be completed and it will be a joint effort with the pediatrics unit from their reference hospital.
